# No Association between Glycemia and Wound Healing in an Experimental *db/db* Mouse Model

**DOI:** 10.1155/2013/307925

**Published:** 2013-10-22

**Authors:** Margrete Berdal, Trond Jenssen

**Affiliations:** ^1^Institute of Clinical Medicine, Faculty of Health Sciences, University of Tromsø, 9037 Tromsø, Norway; ^2^Department of Organ Transplantation, Oslo University Hospital, Rikshospitalet, 0424 Oslo, Norway

## Abstract

Impaired wound healing is a frequent problem in diabetes. Hyperglycemia may be an operative mechanism, but a link between glycemic control and wound healing has never been established. Wounds in *db/db* mice have been extensively studied. This study was undertaken to see if plasma glucose was a predictor of wound healing. An excisional wound was made (149 *db/db* mice). Wound closure was studied versus metabolic variables. The animals were 11.8 ± 0.2 weeks (mean ± standard error of the mean), obese (38.1 ± 0.5 g), and hyperglycemic (fasting plasma glucose 21.0 ± 0.7 mmol/L). Wound closure at day 13 was 30.1 ± 1.6%. In linear mixed model analyses neither fasting plasma glucose nor its change from start to end of experiment was a significant predictor of wound closure (*β* = 0.15, *P* = 0.07, 95% CI: −0.01 to 0.31 and *β* = 0.06, *P* = 0.5, 95% CI: −0.11 to 0.23, resp.). However, increase in body weight significantly and independently predicted wound closure (for weight change, *β* = 0.22, *P* = 0.008, 95% CI: 0.06 to 0.38). This study strongly suggests that wound healing in *db/db* mice is independent of prevailing glycemia but dependent on anabolic changes such as weight gain over time.

## 1. Introduction

Impaired wound healing—a well-known problem in diabetes—has been extensively studied in animals [1, 2]. One model, which has been explored for this purpose, is the genetically leptin receptor deficient, diabetic *db/db* mouse with characteristics such as obesity, transient hyperinsulinemia, insulin resistance, severe hyperglycemia, and impaired wound healing [2–8]. 

Hyperglycemia is one factor that may be implicated in impaired wound repair. Clinical guidelines advocate optimization of metabolic control to facilitate wound healing [9]. A retrospective study in humans suggested that glycated hemoglobin (A1C) predicted healing rates in diabetic wounds [10]. However, as far as we know, direct evidence of a link between glycemic control and healing is lacking [11]. 

In the *db/db* mouse model, a few studies have addressed the possible impact of hyperglycemia on wound healing [6, 12, 13]. They included up to 31 *db/db* mice from one or two age groups, and baseline body weight but not weight change was assessed. No significant associations between metabolic parameters and wound healing were observed [6].

In the present study we included 149 *db/db* mice, aged 6–16 weeks, with the aim of testing whether fasting plasma glucose at baseline, the change in fasting plasma glucose, or other metabolic parameters, including weight change, were associated with wound closure.

## 2. Materials and Methods

### 2.1. Animals

Diabetic C57BL/KsBom-*db/db* mice were studied. All animals were purchased from M&B A/S, Ry, Denmark. The *db/db* strain is a well-recognized model for type 2 diabetes mellitus with an autosomal recessive mutation in the *db*-gene on chromosome 4 and associated deficient leptin receptor [4]. The animals become obese with insulin resistance and hyperinsulinemia. After the age of 2-3 months, atrophy of pancreatic islets causes severe hyperglycemia [3, 15]. 

The animals were housed as previously reported [5]. They were offered rodent food, SDS RM 1 (E) (Special Diets Services, Essex, England) including 2.7% crude oil, 14.4% crude protein, 4.7% crude fibre, and 44.9% starch. Furthermore, the animals had free access to water. Body weight was measured once or twice a week (Mettler PM 2000, Mettler Instrument Corp., Hightstown, NJ, USA). The Norwegian Ethics Committee for Research on Animals approved the experimental protocols.

One hundred and forty-nine* db/db *mice (86 females) from 16 batches were studied. The size of the batches from 1 to 16 was as follows (batch number: *n* mice): 1: 5, 2: 12, 3: 9, 4: 13, 5: 8, 6: 7, 7: 6, 8: 16, 9: 6, 10: 16, 11: 5, 12: 8, 13: 5, 14: 5, 15: 9, and 16: 19. At baseline, the batch wise average body weight (day 7 after wounding) was 32.9–47.1 g, and the average fasting plasma glucose at wounding (day 0) ranged from 9.5 to 29.9 mmol/L. A1C was measured at day 0 in 43 animals from five batches (4, 5, 12, 14, and 15), and the batch means were between 9.1 and 11.8%.

During followup the mean batch wise weight change between days 7 and 13 of experiment varied from −5.6 to +8.1%, the corresponding change in fasting plasma glucose between day 0 and end of experiment varied from −2.9 to +164.4%, the mean batch-wise wound closure rate between days 6 and 13 was between 0.039 and 0.142 cm^2^/day, and the mean wound closure at day 13 ranged from 5.1 to 48.7%. 

The experiments started when the age (weeks) of the animals was as follows (breeder's information): 6–8 (*n* = 12), 7–9 (*n* = 7), 10-11 (*n* = 11), 10–12 (*n* = 5), 10.5–12.5 (*n* = 9), 11–13 (*n* = 33), 12-13 (*n* = 13), 12–14 (*n* = 42), 13-14 (*n* = 4), 13–15 (*n* = 9), and 14–16 (*n* = 4). 

### 2.2. Anesthesia and Blood Sampling

General anesthesia was introduced after 4 hours of fasting (but still with water *ad libitum*) using a mixture of Hypnorm (Janssen Pharmaceutica BV, Beerse, Belgium) and Dormicum (F. Hoffmann-La Roche AG, Basel, Switzerland) (final concentrations: 0.079 mg/mL fentanyl, 2.50 mg/mL fluanisone, and 1.25 mg/mL midazolam; dose: 0.0075 mL/g body weight administered subcutaneously). Blood samples were drawn from the large saphenous vein on anesthetized animals, placed in heparinized tubes, and stored in ice for approximately one hour until the measurements of fasting plasma glucose (fPG), A1C, and plasma lactate (p-lactate). 

### 2.3. Wounding

We used the same excisional model as previously reported, which is a modification of that described by Greenhalgh et al. [2, 5]. Briefly, the procedure was performed on anesthetized animals having the midpart of their back shaved, chemically depilated using Nair cream (Carter-Wallace Ltd., Folkestone, Kent, England), and washed with tap water. A 1.5 × 1.5 cm^2^ area of the skin on the back was then marked using a template, and the depilated area was thereafter disinfected with chlorhexidine 5 mg/mL prepared at the hospital's pharmacy. Finally, the skin was washed with sterile water. 

A full-thickness skin wound was made, under optimally clean conditions, by excising the skin corresponding to the marked area and the panniculus carnosus. The wound was covered with a semipermeable, transparent polyurethane dressing, Opsite Flexigrid (Smith & Nephew Medical Ltd., Hull, England), which was fixed with the tissue adhesive, enbucrilate (Histoacryl, B. Braun Melsungen AG, Melsungen, Germany), and 5–0 Monosof sutures (Auto Suture Company, Norwalk, CT, USA). The wound margins were finally traced onto glass microscope slides (= area day 0). Before complete fixation of the wound dressing, topical placebo treatment (100 *μ*L NaCl 9 mg/mL; Fresenius Kabi Norge AS, Halden, Norway) was injected onto the wound. Then, buprenorphine was given subcutaneously as analgesia (final concentration 0.030 mg/mL buprenorphine; dose: 0.007 mL/g body weight). Another dose of buprenorphine was given 12 hours after the surgical procedure. An isotonic electrolyte solution, Ringer Acetate (Fresenius Kabi Norge AS, Halden, Norway), was given subcutaneously 0 and 2 hours after wounding. 

A total of 149 diabetic *db/db* mice were studied. One hundred and ten of these received topical placebo wound treatment with 100 *μ*L of NaCl 9 mg/mL daily applied for five consecutive days (0–4). The remaining 39 animals had topical treatment with 100 *μ*L of the macrophage stimulant, aminated *β*-1,3-D-glucan (AG) at day 0 only [7]. We chose to include this group since this intervention was similar to topical placebo wound treatment regarding wound closure at day 13 and baseline characteristics (age, body weight, fPG, and p-lactate; all *P* > 0.1, *t*-tests) [7]. Furthermore, this group was similar regarding changes in weight (between days 7 and 13), fasting plasma glucose, and plasma lactate (both of the latter between day 0 and end of experiment; all *P* > 0.1, *t*-tests).

The conditions of the animals during the experiments and the procedures performed at the end of the study were as previously reported [5]. The observation period lasted for 13 to 63 days. All experiments were performed within a time frame of 24 months. Results from 20 *db/db* mice have previously been presented [5, 7]. 

### 2.4. Wound Closure Measurement

The wound margins were traced onto glass microscope slides every second to the fourth day during the experimental period. Wound area measurements at days 0 and 13 were performed by two different methods in all 149 mice:

1. manual method is as previously described [5];
2. digital method: wound tracings were digitized by means of a colour image scanner, Canoscan N656U (Canon Inc., Tokyo, Japan). Wound areas (cm^2^) were then calculated by computerized planimetry using Adobe Acrobat 7.0 Professional (Adobe Systems Inc., San Jose, CA, USA).

Wound closure at day 13 was calculated as the percentage change in area, as previously described, by using the formula where day 0 is the day of wounding [5]:

(1) Wound closure  (%)=(Areaday0−Areaday13)Areaday0×100.

In our previous studies with this mouse model, average wound closure (± SE) at day 13 was 17.9 ± 5.7% in the placebo group, and this time point is on the steepest range of the wound closure curve [5]. 

### 2.5. Comparison of the Methods

**Figure 1:**
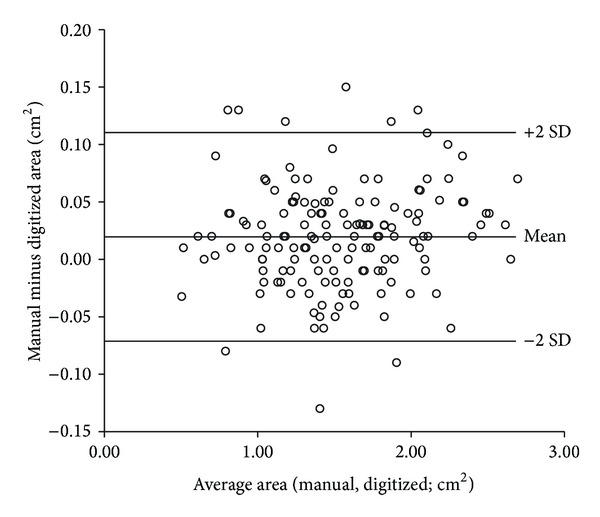
Method comparison. Bland-Altman plot of wound area measurements (day 13) in 149 diabetic *db/db* mice performed by two methods (manual and digitized) [14]. The average of the areas versus the differences between the areas (manual minus digitized) was not correlated, coefficient of correlation adjusted for batch variation, *r* = 0.11 (95% CI −0.03 to +0.25, *P* = 0.1, linear mixed model analysis).

Agreement between the two methods of wound area measurement at day 13 was evaluated by means of a scatter plot (Figure 1) [14]: the average areas (manual, digitized; cm^2^) versus the difference between the areas (manual minus digitized; cm^2^). 

One hundred and forty (94%) out of 149 observations were within the limits of agreement (mean ± 2SD; Figure 1). The coefficients of variation (CV) were 2.2% and 0.50% for the manual and the digitized methods, respectively. Since the digitized method had a lower CV and was more convenient to perform, we chose to report the results of this method in the present study. 

### 2.6. Metabolic Parameters

Fasting plasma glucose and lactate were measured by YSI Glucose and L-Lactate Analyzer Model 2300-GL STAT (Yellow Springs Instrument Co., Yellow Springs, OH, USA). A1C was analysed using the DCA 2000+ Analyzer Model 5031 C (Bayer Corporation, Elkhart, IN, USA). The analyses were performed according to the manufacturer's guidelines.

### 2.7. Bacteriological Examination and Fungus Cultivation

Samples were harvested from wound bed abradant on anesthetized animals at the end of the experimental period. Animals with signs of wound infection (green-yellowish secretion and decreased closure rate) and/or growth of wound pathogens (e.g., *Staphylococcus aureus*) were excluded from the study.

Among 154 mice five (3.2%) were excluded since they had signs of a wound infection. Specimens for bacterial growth were taken from two of these wounds. In one *Staphylococcus aureus* was detected, and in the other abundant growth of *Klebsiella pneumoniae* was found. 

### 2.8. Statistical Analysis

The data distributions were evaluated by visual inspection of frequency histograms as well as tests of normality, linearity, and equality of variances. Normally distributed data are presented as mean ± standard error of the mean (SE), and statistical significance between groups was tested by *t*-tests. Skewed data were analyzed by Mann-Whitney *U* test. 

Heterogeneous variances for all variables were present between the 16 batches of animals and warranted the use of linear mixed model (LMM) analyses to evaluate the independent relationship between the variables studied. The percentage wound closure at day 13 after wounding (wcl_day13_) was the dependent variable. The fixed factors were age_day0_, fPG_day0_, and p-lactate_day0_ at wounding (day 0), body weight at day 7 (wt_day7_), the percentage body weight change between days 7 and 13 after wounding (Δwt_day7−13_), and the percentage change in fPG during the experimental period from baseline to cervical dislocation (from day 0 to the end; ΔfPG_day0−end_). No other blood samples were drawn before sacrifice. Batch was specified as a random factor, and a diagonal covariance structure was selected for the residuals of the random factor.

Age_day0_was estimated as a mean of the age interval indicated by the breeder. The points of time for Δwt_day7−13_-measurements (days 7 and 13) were chosen to represent, as much as possible, the degree of wound closure between days 0 and 13 (wcl_day13_) and to avoid effects of potential stress reactions associated with the wounding. For the same purpose fPG was measured before wounding at day 0 (fPG_day0_; see Section 2.2). Potential interaction effects were tested by including the product of the variables in the models. Statistical analysis was performed by the software IBM SPSS, version 19.0 (IBM Corp., New York, NY, USA). *P* < 0.05 (2-tailed) was considered statistically significant.

## 3. Results

### 3.1. Characteristics and Wound Healing in the Experimental Animals

At baseline all mice (*n* = 149) had developed obesity and polyuria, characteristics consistent with diabetes. Analyses demonstrated increased levels of fPG_day0_, p-lactate_day0_, and A1C_day0_ (Table 1) [5]. Baseline and followup data (weight change between days 7 and 13, changes in fPG and A1C between day 0 and end of experiment, and wound closure at day 13) of the animals did not differ between females and males (*P* > 0.07 for all comparisons) and were consequently pooled and analyzed together.

Fasting PG_day0_ demonstrated a highly significant, batch-adjusted correlation with A1C_day0_ (*n* = 43, *r* = 0.65, *P* < 0.0005, and 95% CI 0.48–0.81). Correspondingly, ΔfPG_day0−end_ was significantly associated with the change in A1C during the experiment (from day 0 to the end, ΔA1C_day0−end_; *n* = 43, *r* = 0.62, *P* < 0.0005, 95% CI 0.44–0.81, linear mixed model analyses). 

The followup data in all mice were as follows: change in body weight between days 7 and 13 was −0.9 ± 0.5% (mean ± SE), change in fPG from day 0 to end of experiment was 24.6% (−86.6 to 416.7%) (median (range)), wound closure rate between days six and thirteen was 0.097 ± 0.005 cm^2^/day, and wound closure at day 13 was 30.1 ± 1.6%.

### 3.2. Wound Healing in Diabetic *db/db* Mice: Linear Mixed Model Analysis

Age, metabolic variables, and wound closure 13 days after wounding in 149 diabetic *db/db* mice were studied. LMM analyses showed that the change in body weight between days 7 and 13 after wounding was a significant and independent predictor of wcl_day13_ (Table 2). Baseline age, weight, fasting plasma glucose, and lactate as well as the change in fPG from day 0 to end of the experiments, however, were not associated with wound healing (Table 2). 

A1C at wounding (day 0) and the change in A1C between day 0 and the experimental ending were measured in a subgroup of animals (*n* = 43). In a separate model with analysis of data from these 43 animals, A1C_day0_ predicted wcl_day13_ when adjusted for age_day0_,  wt_day7_, Δwt_day7−13_, ΔA1C_day0−end_, and p-lactate_day0_ (*β* = 0.36, *P* = 0.003, and 95% CI 0.14–0.58). In this model age_day0_, as well, appeared as a significant predictor (*β* = 0.28, *P* = 0.01, and 95% CI 0.08–0.48). These predictors were not correlated with each other (*r* = −0.14, *P* = 0.3), and there was no interaction between them (age_day0_ × A1C_day0_, *P* = 0.5).

### 3.3. Wound Healing in Diabetic *db/db* Mice Gaining or Losing Weight

Among the animals, 61 (41%) gained weight, and 88 (59%) lost weight (wt change on average +4.6 ± 0.5% and −4.8 ± 0.3%, resp.) between days 7 and 13 of the experiment. At baseline the group of animals gaining weight was younger and weighed less than the animals losing weight, age: 10.9 ±  0.3 versus 12.4 ± 0.1 weeks, *P* < 0.0005; weight: 36.5 ± 0.8 versus 39.3 ± 0.6 g, *P* = 0.01. Mice gaining weight had significantly lower fPG_day0_ than those losing weight (14.7 ± 0.9 versus 25.4 ± 0.8 mmol/L, *P* < 0.0005). The corresponding numbers for A1C_day0_ were 7.7 ± 0.5  (*n* = 9) versus 10.6 ± 0.3% (*n* = 34), respectively, *P* < 0.0005.

**Figure 2:**
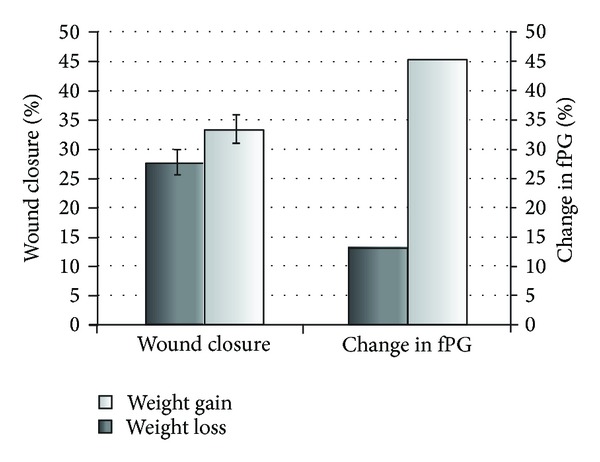
Wound closure at day 13 and the change in fasting plasma glucose between start (day 0) and end of the experiment (ΔfPG_day0−end_) in *db/db* mice gaining (*n* = 88) or losing (*n* = 61) weight from day 7 to 13. Wound closure (mean ± SE) was significantly higher in animals gaining weight, compared to those losing weight, standardized estimate of the difference with multivariate adjustment, *β* = 0.29, *P* = 0.015, and 95% CI 0.07–0.51 (linear mixed model analysis). Correspondingly, ΔfPG_day0−end_ was significantly higher in the group gaining weight versus the one losing weight, 45.6 (−76.2 to +416.7)% versus 13.2 (−86.6 to +181.8)% (median (range)), *P* < 0.0005, Mann-Whitney *U* test.

LMM analysis with adjustment for age_day0_, wt_day7_, fPG_day0_, ΔfPG_day0−end_, and p-lactate_day0_ demonstrated significantly higher wcl_day13_ in animals gaining weight, compared to those losing weight (Figure 2). However, fPG_day0_ and ΔfPG_day0−end_ did not predict wcl_day13_ in this analysis (*P* ≥ 0.1 for both estimates). 

## 4. Discussion 

The objectives of our experiments were to determine if there was a relationship, first, between glycemia and wound healing and, second, between other metabolic variables and wound healing in diabetic *db/db *mice. 

Baseline fasting PG and the change in fPG during followup (experimental start to the end) did not predict wound closure at day 13. However, change in body weight between days 7 and 13 after wounding was significantly and independently associated with wcl_day13_, and wound closure was significantly higher in animals gaining weight compared to those losing weight (Figure 2). 

Two other wound models have failed to show any association between wound closure and fasting blood glucose [6, 12]. Blood glucose, not plasma glucose, was measured in these studies. Our study failed to show any association between wound healing and change in plasma glucose over time. Furthermore, Pietramaggiori and coworkers examined 4-week-old female *db/db* mice after being exposed to peripheral blood circulation from age-matched nondiabetic (*db/+*) littermates through parabiotic joining. Thirty days later, the diabetic animals (*db*-chimera) showed significantly improved wound healing compared to controls in spite of prevailing hyperglycemia [8]. These wounds demonstrated increased presence of macrophages and T lymphocytes. Furthermore, at most 20% of the circulating cells were derived from the nondiabetic partner [8]. This suggests that improved wound healing in this model was related to normalization of circulating leukocyte frequencies and improvement in inflammatory markers. 

We have previously found that insulin treatment in *db/db* mice does not result in significant improvement in wound healing despite significant reductions in plasma glucose levels [5]. 

No significant correlations between weight change and degree of wound closure have been reported before [6]. However, in 6-week-old mice of this strain, beta-3 adrenoceptor agonist treatment was associated with normalized healing of excisional wounds and maintenance of the body weight as compared to controls showing reduced weight [12]. On the other hand, corresponding studies with beta-3 adrenoceptor agonist treatment in 17-week-old animals revealed no significant difference regarding wound closure in the same model. The body weight of the animals was not reported [12]. 

Guidelines for the treatment of diabetic wounds in humans claim optimization of metabolic control [9]. The burden of proof is disputable, since direct evidence of a link between glycemic control and healing is lacking [11]. A retrospective study in humans suggests, however, that A1C predicts healing rate in diabetic wounds [10]. 

In our model neither fasting plasma glucose at baseline nor the change in fasting plasma glucose from start to end of the experiment predicted wound closure. 

Since the linear mixed model analysis procedure did not include the calculation of percentage explained variance for these variables, we used an ordinary multiple linear regression analysis showing that fPG_day0_ and ΔfPG_day0–end_ accounted for 0.8% and 0.1% of the total variation in the model, respectively. Weight change accounted for 1.4% of the variation. However, this procedure did not adjust for heterogeneous variances between the batches and correlation within the batches of animals. Thus, these are rough estimates only.

LMM analysis of subgroup data (*n* = 43) revealed both A1C_day0_ and age_day0_, but not Δwt_day7−13_, as significant and positive predictors of wound closure at day 13. This could either mean that A1C is a more powerful predictor of wound closure than plasma glucose concentrations or that this is caused by chance in relation to sample selection (sampling error). 

Weight change, a significant and independent predictor of wound closure in our model, also demonstrated a negative association with plasma glucose (Δwt_day7−13_ versus fPG_day0_, *r* = −0.54, *P* < 0.0005, and 95% CI −0.65 to −0.42, LMM analysis). Consequently, loss of body weight tended to occur in the most severe hyperglycemic mice, which could be expected, since weight loss represents a negative caloric balance [15, 16]. Weight gain may reflect hyperphagia due to deficient regulation of satiety and/or increased metabolic efficiency, presumably related to hyperinsulinemia [3, 4].

Loss of calories may first be related to glucose-associated osmotic diuresis. Urine output has been reported to exceed the water intake in these animals [17]. Second, lipid metabolism during the natural course of the syndrome may be implicated since decreased lipogenesis is present in the later stage [15]. Other studies on wound healing in the *db/db* model reported a comparable weight loss [12, 18]. 

Nutrition is important to wound healing since the process of repair requires energy from carbohydrates and fat as well as proteins for collagen synthesis [18]. We wanted to study diabetic mice without certain diet restrictions and therefore chose to feed all mice a standard rodent diet (see Section 2). This diet appeared to be in accordance with the maintenancediet for adult rodents (AIN-93 M), recommended by the American Institute of Nutrition [19]. We, therefore, assumed that consumption of this diet was sufficient to meet the nutritional needs of our animals, including the needs for wound repair. 

Our experiments did not show any association between age_day0_ and wcl_day13_. This is in contrast to other studies demonstrating impaired wound healing in older compared to younger *db/db *mice [13, 20]. However, these studies reported several months' difference between younger and older age groups, so the age span in our animals may have been too narrow to show any relationship to wound closure [13, 20]. 

The strengths of our study include a higher number of experimental animals (*n* = 149) compared to others [6, 12, 13]. Furthermore, our experimental animals had a wide range of fasting plasma glucose levels, and we controlled not only for baseline glycemia, but also for change in glycemia during followup. Another advantage of our study was a thorough validation of the wound area measurement method (Figure 1). 

Limitations are also present in our study. First, food- and -water intake as well as diuresis was not measured. This is, in particular, a disadvantage in relation to the observed weight change. Second, a measurement of PG represents a certain point in time on a fluctuating curve and may deviate significantly from the average PG level [21]. Multiple measurements over time may differ and contribute to the relatively large intraindividual biological variability also known in humans, especially during periods of stress and illness [22, 23]. 

In conclusion, our studies demonstrate that neither fPG_day0_ nor ΔfPG_day0−end_ is a significant predictor of wcl_day13_ in diabetic *db/db* mice. However, weight gain during followup is associated with subsequent wound closure.

In planning future experiments on wound healing in *db/db* mice, catabolic changes may be more important to address than glycemia.

## Figures and Tables

**Table 1:** Baseline characteristics of the experimental animals.

Characteristic	Mean ± SE
Age_day0_ (weeks)	11.8 ± 0.2
Body weight_day7_ (g)	38.1 ± 0.5
fPG_day0_ (mmol/L)	21.0 ± 0.7
A1C_day0_ (%)*	9.9 ± 0.3
p-Lactate_day0_ (mmol/L)	1.6 ± 0.04

Characteristics of 149 (86 females) diabetic *db/db* mice. Baseline body weight was measured seven days after wounding (day 7) to avoid effects of potential stress reactions associated with the wounding. *n = 43. Day 0: the day of wounding; fPG: fasting (4 hours) plasma glucose; A1C: glycated hemoglobin; p-lactate: plasma lactate.

**Table 2:** Adjusted estimates for age and metabolic variables from 149 diabetic *db/db* mice in linear mixed model (LMM) analyses with wound closure at day 13 as the dependent variable.

Predictor variables	Standardized estimate	*P* value	95% CI for the estimate
age_day0_ (weeks)	0.06	0.71	−0.25 to 0.36
wt_day7_ (g)	−0.06	0.49	−0.25 to 0.12
wt_day7–13_ (%)	0.22	0.008	0.06 to 0.38
fPG_day0_ (mmol/L)	0.15	0.07	−0.01 to 0.31
ΔfPG_day0–end_ (%)	0.06	0.47	−0.11 to 0.23
p-Lactate_day0_ (mmol/L)	−0.02	0.69	−0.14 to 0.09

The model was adjusted for the variation between batches of animals by means of LMM. Tests of potential interactions (Δwt_day7–13_ × fPG_day0_, Δwt_day7–13_  ×  ΔfPG_day0–end_) adjusted for age_day0_, wt_day7_, Δwt_day7–13_, fPG_day0_, ΔfPG_day0-end_, and plasma lactate_day0_ were nonsignificant (*β* = 0.04, *P* = 0.85, and 95% CI −0.35 to 0.42; *β* = −0.08, *P* = 0.34, and 95% CI −0.25 to 0.09, resp.). Day 0: the day of wounding; wt_day7_: body weight 7 days after wounding; Δwt_day7–13_: body weight change between days 7 and 13; fPG: fasting plasma glucose; ΔfPG_day0–end_: the change in fPG from day 0 to the end of experiment; p-lactate: plasma lactate.
